# Chromatid recommensuration after segmental duplication

**DOI:** 10.1186/1742-4682-6-1

**Published:** 2009-01-07

**Authors:** Mark N Jabbour

**Affiliations:** 1 Department of Pathology, American University of Beirut Medical Center, Beirut, Lebanon

## Abstract

**Background:**

Midsegment duplication (dup) of chromatid arms may be symmetric or asymmetric. It can be argued that every dup should yield a discommensured RC with (a) loss of at least one duplicated unit to the template counterpart and; (b) deletion of all sections of the replicating chromatid arm that are distal to both the gap left by the duplicating process and the segment closest to the centromere.

**Hypothesis:**

Mechanisms capable of recommensuring the stack of chromatids after topological shifts of duplicated units (dups) are discussed. The mechanics might fail in few cases, which are discussed in terms of statistics and scalability.

**Conclusion:**

The dynamics of the highly non-linear processes discussed here may be relevant to duplications of smaller (epsilon) subunits such as telomeric units within malignant genomes.

## Background

Midsegment duplication (dup) of chromatid arms has been discussed in [1]. Alternative formats of the process yield duplicated units (dups) that are either "direct", i.e. stacked congruently in the chromatid, or "indirect", i.e. stacked upside down along the arm of the hosting chromatid. Indirect dups are rarer [2]. Unlike deletions, dups occur infrequently within malignant cells during replication of sister chromatids (RC) from their template counterparts (TC). The schematics (panels 2, 2a and 2b in [1]) show how the TC appropriates a segmental unit (dup 5, black) from the RC, in which a gap is left when dup 5, the missing unit, is captured by the TC. The series of compressions and expansions to which the chromatid is subjected to by shock-waves traveling through the cytoskeleton and nuclear scaffold result in a discommensured TC. Subsequently, the TC captures and intercalates dup 5 from RC in either a symmetrical (panel 2a in  [1]) or asymmetrical (panel 2a' in  [1]) fashion (symmetry refers to the points of attachment of the duplicated units to the TC). Moreover, in both the symmetric and asymmetric fashion, TC acquires a dups that contributes to the overall discommensured state. After recommensuration (see below) the dup 5 unit is incorporated into the TC (panel 2b in  [1]), while the asymmetric incorporation of dup 5 results in either intercalation with deletion of the extra segment or deletion of the whole arm (panel 2b' in  [1]). Generalizing, every dup should invariably yield a discommensured RC with two concomitant defects: (a) loss to the TC of one (or more) duplicated unit; (b) deletion of all RC arm sections that are distal to both the gap and the segment closest to the centromere.

This paper discusses how: (a) the occurrence of RC deletions might be reduced notwithstanding the capture of cuts and gaps from dup units captured by sister TC; (b) recommensuration of TC might ensue once a dups is captured in a symmetrical and asymmetrical fashion. The discommensuration, manifested as a solitonic kink/loop, is annihilated by a series of compressions, gyrorotations and expansions during either eversion or topological rectifications of their defective chromatid stack.

## Avoidance of deletion by gap closure of the RC stack

**Figure 1:**
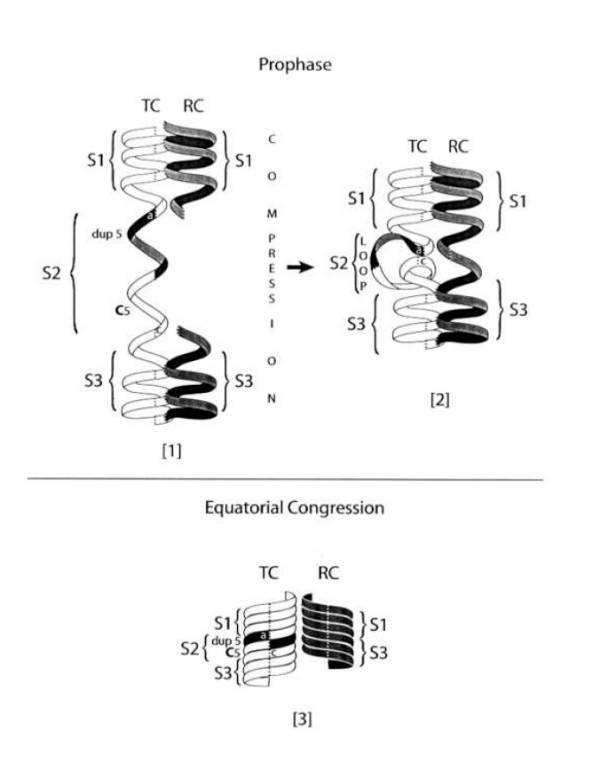
**Panels (1, 2) depict intranuclear events that close the RC gap at prophase**. Panel (3) illustrates rectification of the TC stack as TC and RC congress to the metaphase equator, in a (re)commensured state. For additional details, see text.

Panel (1) of Fig. 1 reproduces panel 2a of [1] with a few modifications such as a slight rotation of the template chromatid arm (TC), with segments (S) identified as shown. S2 carries dup 5 (black) coaxially stacked above unit C5. Abutted to TC are segments S1 and S3 of replica chromatid RC, omitted in [1]. These segments are initially separated by the gap left when dup 5 is incorporated by the TC. Presumably, such configurations occur at prophase when the still-intact nuclear envelope allows "last minute" repair of chromatid defects before its breakdown limits rectification of chromosomes traveling to equatorial congression. Besides occasioning eversion (a process that involves disengagement of the template chromatid from the replica by turning inside out, producing two coiled sister chromatids with opposite chiralities) of RC from the TC in the typical side-by-side arrangements of panel (1), late prophase initiates compression of the chromatids into the more condensed state of panel (2). Herein we consider only compressive actions that compact the TC, RC and segments thereof. Compaction might be synergized during condensation of neighboring chromatids and/or localized nuclear contractions (nucleostalsis).

The anomalous behavior of the TC is of interest. In contrast to S1 and S3, segment S2 is not flanked by its replica from the RC. During compression, the "unsupported" S2 is liable to deform into the (C5-dup5) loop and to flex out of alignment, as in panel (2). Misalignment abuts S1 to S3, thus closing the S1–S3 gap on the RC, a precondition for reconnecting its severed stack before the end of prophase. After nuclear envelope breakdown, the RC should emerge as a normally conformed coil despite the loss of the C5 unit. Being coaxially straight, RC is streamlined to minimize the viscous drag from the spindle's fibrogel, which impacts on every chromatid traveling to equatorial congression. As arm separation between the chromatids becomes more pronounced (panel (3)), the equatorial approach of any chromatid becomes progressively less affected by the kinematics of the arms of its sister. Aside from restoring commensuration and physical integrity to the RC, the dynamic evolution of the TC is by no means trivial because of the deforming loop S2 schematized in panel (2). The loop distorts the TC so that its stack is not optimally streamlined to minimize viscous drag during equatorial congression through the spindle's fibrogel. To reduce such drag, S2 must be rewound and restacked coaxially, as in panel (3). Such topological rectifications may be chirally prompted by two synergistic processes. One is the screw-like gyrorotation from segments S1 and S3 as they confront the spindle viscosity. In addition to attenuating flailing movement by keeping the TC vectorially straight, gyrorotation may suffice to rewind S2 into the chirally commensured and coaxially stacked segment in panel (3). The second component stems from the spindle's physico-chemical environment. This compacts the chromatid arms significantly more than during prophase. Thanks to these corrections, S2 is stabilized as a regular coil sandwiched between S1 and S3. Eventually, the initially suboptimal aspect-ratio of the TC (panels 1 & 2) changes, as in panel (3), thus reducing viscous drag not only en route to equatorial congression but also in preparation for the chromatid's return trip to the reconstituting nucleus during telophase.

Although it will tend to reduce deletions, the above process might perturb the TC in various topological and structural ways. A few examples are discussed here without diagrams. In panel (2), loop S2 connects S1 and S3 by links *a *and *c*. Located at the "neck" of the loop, these links are almost contiguous, so S2 might erroneously link S1 at *c *to S3 at *a*. Such an inverted configuration succeeds only if *a *and *c *rotate 180^0 ^orthogonally to the long axis of the TC. Rotations of that kind are unusual because it is relatively unlikely that S2 will connect to *c *at S1 and *a *at S3 to form an inverted cross-linked loop at *a *and *c*, a process that would require a higher kinetic energy than the S2 loop formed in panel (2). Consequently, indirect or upside-down duplications are less probable than the direct variants.

## Conclusion

The chromatin is quite flexible during prophase. We may predict that S2 might loop-out one or both subsegments, so indirect duplications might involve one or both units, either C5 or dup 5. If links *a *and *c *do not undergo 180^0 ^rotation, *a *might connect to *c*, thus organizing a dup5-C5 (ring-like) split minichromatid that is multiduplicated and partitioned erratically through successive mitoses, whereas the TC either retains its full S1–S3 complement or is shortened by segmental losses. In other words, the multiduplicated minichromatid is subjected to enzymatic cleavage and may subsequently be lost from the TC. Referring to [3,4] and references therein for technicalities, the final orientation of dup 5 in panel (3) is equivalent to C5. This means that genes on the hull of both units are identically accessible to transcription. This is in contrast to genes on the RC, unless subverting entanglements alter the topology so that the hull segment permutes towards the inner facet rendering it inaccessible to transcription factors and subsequent gene expression. Finally, the dynamics of these highly non-linear processes may be relevant to duplications of smaller (epsilon) subunits into which the chromatid architecture is scalable in regards to both normal and pathological processes such as malignant genomes either prior to or after therapy [5].

## Competing interests

The authors declare that they have no competing interests.
